# Resolution of Pneumomediastinum, Cervicofacial Emphysema, and Internal Ophthalmoplegia with Hyperbaric Oxygen Therapy After Hip Arthroscopy: A Case Report

**DOI:** 10.3390/jcm15124634

**Published:** 2026-06-15

**Authors:** Samuel J. Thomas, Jeffery M. Bao, Aida Ansari, Eshaal Kizilbash, Jack H. Langford, Nicholas E. Kalafatis, Aumer Shughoury, Diane M. Gregory, John R. Larson, Nicholas J. Adams, Mathew K. Marsee, Christopher C. Jordan, David M. Johnson, Syed Raza, Ann Wiarda, Adeela M. Alizai, Mark M. Walsh

**Affiliations:** 1 Department of Emergency Medicine, Indiana University School of Medicine, South Bend, IN 46617, USA; 2 Department of Emergency Medicine, Saint Joseph Regional Medical Center, Mishawaka, IN 46545, USA; 3 Department of Medicine, Royal College of Surgeons in Ireland, D02 YN77 Dublin, Ireland; 4 Department of Ophthalmology, Indiana University School of Medicine, Indianapolis, IN 46202, USA; 5 Department of Wound Healing, Saint Joseph Regional Medical Center, Mishawaka, IN 46545, USA; 6 Department of Sports Medicine, Saint Joseph Regional Medical Center, Mishawaka, IN 46545, USA; 7 Department of Critical Care Medicine, Saint Joseph Regional Medical Center, Mishawaka, IN 46545, USA; 8 Department of Ophthalmology, Grossnickle Eye Center, Mishawaka, IN 46545, USA; 9 Department of Medical Ophthalmology, Michiana Ophthalmology and Headache, Mishawaka, IN 46545, USA

**Keywords:** arthroscopy, subcutaneous emphysema, internal ophthalmoplegia, hyperbaric oxygenation, case report

## Abstract

**Background/Objectives**: Hip arthroscopy is a minimally invasive procedure with rare complications that can occur due to air entry outside the joint space. **Case Presentation**: A 19-year-old patient underwent right hip arthroscopy with attempted joint venting. The next morning, she had pain in her right leg, neck, and chest with paresthesias over her hands and feet. A subsequent emergency department physical exam revealed crepitation of the lower extremities, abdomen, chest, and neck caused by air entrance during arthroscopy. The patient also reported blurred near vision. Additionally, the pupils were fixed, did not accommodate, and were dilated at 7 mm. Computed tomography scans revealed subcutaneous emphysema, pneumoperitoneum, pneumomediastinum, and cervicofacial emphysema. Magnetic resonance imaging of the brain revealed a Chiari I malformation. The patient received four hyperbaric oxygen treatments. By the fourth treatment, near visual acuity had improved, but far visual acuity had worsened. Vision had returned to normal eight days after discharge. **Conclusions**: It is proposed that the patient’s reduced near vision, accommodation paralysis, and fixed and dilated pupils were brought about by pneumomediastinum and cervicofacial emphysema, inhibiting the ability of the pupils to constrict, causing bilateral mydriasis and accommodation paralysis for near targets. Additionally, the subsequent transient myopic shift is a known complication of hyperbaric oxygen therapy, which increases the refractive index of the crystalline lens.

## 1. Introduction

Hip arthroscopy is a minimally invasive procedure that can be performed to treat many different hip conditions. Complications arising from hip arthroscopy are rare, with an estimated complication rate of 1 to 8%, the majority of which are not life-threatening [1]. The surgery requires ample space for the arthroscope and other instruments, and this space can be obtained using irrigating fluids, such as saline, which separate the acetabulum from the femoral head, allow adequate exposure of the joint capsule to the instruments, and improve visualization by clearing debris from the joint [2,3]. In the past, air and other gases were often used to distend joints during arthroscopy [4]. However, now it is less common for air to be used for joint distension, since attempting to inject air into the joint can lead to air embolism if air gets into the vasculature, and fluids are more effective in distending the joint [5,6]. Injection of air into the joint is still used in arthroscopies for joint venting, a method of decreasing the traction force necessary to distract the hip by breaking the joint’s negative pressure seal [7,8].

In addition to air embolism, arthroscopic surgeries have been known to cause subcutaneous emphysema and pneumomediastinum after the entrance of air outside the joint [9,10]. Hyperbaric oxygen therapy (HBOT) is a recommended treatment for both air embolism and severe subcutaneous emphysema [11,12]. The rationale of using HBOT for these gas-related complications is that increased pressure will reduce the volume of the gas according to Boyle’s Law, and breathing 100% oxygen increases blood and tissue oxygenation [12,13].

We present a case of massive entrance of air into the tissues as a complication of injected air during hip arthroscopy for a patient who developed symptoms including crepitation, swelling, pain, and loss of visual accommodation. This case highlights the problems that can occur after hip arthroscopy and how treatment with HBOT was associated with improvement of symptoms after four days. This case report draws attention to the effects of the entrance of air outside its normal anatomic space. Specifically, this patient presented with concerns about numbness and tingling, and a careful examination revealed subcutaneous emphysema not only in the lower extremities but also above the diaphragm. This led the physician to ask questions about neurological symptoms, thereby uncovering the visual disturbances.

## 2. Case Presentation

A 19-year-old female cross-country runner underwent her second arthroscopy of the right hip due to adhesive capsulitis of the hip and labrum tears that failed to respond to the first attempt six months earlier. During the surgery, the patient received general anesthesia in a supine position. The patient underwent labral repair, acetabuloplasty, femoroplasty, capsular plication, and debridement of capsulolabral adhesions. Because of the scarring associated with the previous labrum repair, she received multiple air venting injections with a syringe in an attempt to break the joint seal and ease the force required to distract the hip. The patient then received fluoroscopy-guided saline irrigation from an automated arthroscopic pump through a 17-gauge needle to successfully distend the joint. No anticholinergic medications or muscle relaxants were administered. After postoperative evaluation, the patient was discharged and traveled four hours back to her home in a sitting position. The next morning, the patient noted pain in her right leg, neck, and chest, along with numbness and tingling over her hands and feet. Therefore, she presented to the emergency department for evaluation.

Upon examination, vital signs were normal. The physical exam revealed crepitation of the lower extremities, abdomen, chest, and neck with swelling of the right leg, chest, neck, and face. The patient also reported an inability to read her phone up close, but distance vision was unimpaired. Corrected visual acuity was 20/20 bilaterally at distance but only 20/100 bilaterally at near, as measured with a Snellen chart. In addition, the pupils were fixed and dilated at 7 mm, unreactive to light, and did not accommodate to near fixation. The patient had no history of ophthalmological diseases and wore glasses for distance vision. The conjunctiva and sclera were normal. Slit lamp examination revealed normal corneas, anterior chambers, and irides. Funduscopic evaluation revealed normal optic nerve, macula, and vasculature in both eyes. The artery vein discs were sharp, and the macula was normal. Laboratory results were normal, but computed tomography (CT) scans of the abdomen and pelvis, chest, and neck revealed massive subcutaneous emphysema involving the right hip, abdomen, chest, and neck, with pneumoperitoneum and pneumomediastinum (Figure 1). A head CT showed no air in the brain or orbits. Likewise, magnetic resonance imaging (MRI) of the brain showed no intracerebral air but did reveal the presence of a 7-mm Chiari I malformation (Figure 2).

After consultation with an HBOT expert, the patient received four treatments using the United States Navy Table 6 dive protocol (Figure 3) [14]. Each HBOT treatment lasted 5 h, with 15 min to reach a maximum pressure of 2.5 atmospheres absolute and 30 min to decompress to ambient pressure. The patient was given five air breaks during the first treatment and two air breaks during the three other sessions. Repeat CT scans on the day of the third treatment revealed the disappearance of the subcutaneous emphysema, pneumoperitoneum, and pneumomediastinum. After the third treatment, the patient’s paresthesias had diminished. After the fourth, no skin crepitation was observed.

However, while near visual acuity gradually returned to 20/30 by the fourth and final day of HBOT, distance visual acuity declined to 20/50. Evaluation by an ophthalmologist revealed a completely normal eye exam (20/20 bilateral vision) 14 days after surgery and 8 days after discharge from the emergency department. The patient was able to return to school, but paresthesias in the right and left calves and upper right thigh continued for about a month after discharge from the emergency department before resolving with no further treatment.

## 3. Discussion

This case details a rare but consequential complication of hip arthroscopy. Subcutaneous emphysema and pneumomediastinum after arthroscopy have been reported in the literature, usually after shoulder arthroscopy [9,10]. In this case, subcutaneous emphysema was likely caused by the entry of air outside the joint space and into the tissues of the hip during joint venting. This may have occurred when air escaped from the joint space before the capsule could be plicated at the end of the surgery. Therefore, careful monitoring of air injections during hip arthroscopy can prevent the occurrence of these complications. From the hip, the air rose through the tissues and musculature into the peritoneum, mediastinum, and neck while the patient was sitting up, resulting in blurred near vision and mydriasis due to bilateral internal ophthalmoplegia, or paralysis of the iris sphincter and ciliary muscles. Visual problems after hip arthroscopy have been reported in the literature as a result of paradoxical air emboli that crossed from venous to arterial circulation and localized to the central retinal artery of one eye [15]. However, this patient was unique since her visual disturbances were bilateral and were likely caused by internal ophthalmoplegia due to pneumomediastinum and cervicofacial emphysema.

This patient might have been more susceptible to develop problems with her vision after entry of air into the cervicofacial tissues because of her pre-existing Chiari I malformation, which has been known to cause blurred vision due to reduced cerebrospinal fluid flow and increased intracranial pressure (ICP) [16]. It is proposed that the patient’s Chiari I malformation and cervicofacial emphysema near the skull base increased ICP. The air in the neck may have increased ICP, similar to a Valsalva maneuver, which has been shown in the literature to exacerbate vision problems in patients with a Chiari I malformation by increasing ICP [17,18]. Increased ICP could have many potential consequences, including an increase in pressure in the interpeduncular and oculomotor cisterns, which surround the oculomotor nerve [19,20]. Increased pressure on the oculomotor nerves could inhibit their function and the function of the iris sphincter and ciliary muscles, which are innervated by the parasympathetic fibers of the oculomotor nerve, causing an inability of the pupils to constrict and resulting in accommodation paralysis for near target [21,22]. Even though the oculomotor nerves are also responsible for eye movement, which was not greatly affected in this patient, it is still possible that the oculomotor nerves were affected by the increased ICP, since the parasympathetic fibers of the oculomotor nerve lie superficially on the cisternal portion of the nerve compared to the deeper somatomotor fibers that control eye movement [21,23]. This increased pressure associated with a Chiari I malformation as a cause for parasympathetic nerve inhibition, resulting in an inability to constrict the pupils, is speculative, and it is perhaps more likely that stimulation of the sympathetic nervous system, which supplies the irides, caused the patient’s mydriasis and inability to accommodate.

Specifically, a more likely cause of bilateral mydriasis is that the pneumomediastinum and cervicofacial emphysema caused irritation and stimulation along the oculosympathetic chain, which is responsible for pupil dilation. The first-order neurons in this pathway descend from the hypothalamus through the brainstem to the cilio-spinal center of Budge near the lungs. The second-order neurons then exit the spinal cord and ascend to the superior cervical ganglia near the skull base. Third-order neurons travel up the neck to the orbits to stimulate the iris dilator muscles and cause pupil dilation [22]. Thus, these nerves, which travel through the chest and neck, may have been stimulated by the air in the mediastinum and neck and caused bilateral mydriasis [24].

Other potential causes of bilateral mydriasis have been explored. The administration of anticholinergic medication, such as a scopolamine patch for nausea, in the perioperative period can cause unilateral mydriasis if the anticholinergic comes into contact with the eye [25]. However, this patient did not receive any anticholinergic medication. Mydriasis related to general anesthesia is usually unilateral, though bilateral cases have been reported. Mydriasis in these cases is usually a function of the adjunctive anticholinergic medicines that are used during anesthesia, so it is unlikely that this patient’s bilateral mydriasis was caused by general anesthesia [26,27]. Therefore, it is unlikely that the bilateral mydriasis was related to pharmacology. Additionally, other mechanisms of interference with pupil constriction were considered, such as the possibility of air entering the ocular orbits. In this hypothesis, air in the orbits may prevent pupil constriction by increasing intraocular pressure, causing optic nerve dysfunction, and interfering with ciliary muscle function [28]. Likewise, air embolism in the brain or ophthalmic arteries was considered as it could cause pupillary complications, such as mydriasis [15,29,30]. However, no evidence of air was detected in the orbits, brain, or ophthalmic arteries on CT, MRI, or funduscopic evaluation. Therefore, it is more likely that the air, without entering the brain or ocular orbits, interfered with the normal function of the parasympathetic or sympathetic nerves that innervate the pupils.

However, all these proposed theories for accommodation paralysis are speculative and cannot be definitively confirmed. The authors theorize that air was the cause of bilateral mydriasis, since there was simultaneous disappearance of the air and reduction in the size of the dilated pupils in response to HBOT. The main limitation of the proposed theories is that this a is a single-case observation from which these conclusions are being drawn. Thus, this case report remains descriptive and hypothesis-generating, since the development of bilateral mydriasis with a loss of accommodation after an arthroscopic surgery has not been described in the literature before, and the mechanisms that caused these symptoms are still unclear.

In this case, HBOT was the treatment of choice for emphysema, since the increased environmental pressure decreases the volume of the subcutaneous emphysema, pneumoperitoneum, and pneumomediastinum, which relieves the pressure on surrounding tissues and organs, and breathing 100% oxygen helps the body to absorb the air into the tissues and further reduces the size of the air pockets [12,13]. After HBOT, the patient experienced an improvement in near vision, likely due to the effectiveness of HBOT in reducing the volume of the pneumomediastinum and cervicofacial emphysema, but she also experienced a decline in distance vision. This myopic shift is a known complication of HBOT caused by oxidative stress in the proteins of the crystalline lens. Since the refractive index of the lens is dependent on the concentration of these proteins, a lower concentration of lens proteins would increase refractive index and induce a myopic shift [31,32,33]. The transient decline in distance vision had resolved eight days after HBOT was completed.

Although HBOT led to successful resolution of air emphysema in this patient and the eventual resolution of symptoms, the trade-off between the risks and benefits of HBOT, along with the severity of the air emphysema, should be considered. HBOT carries many potential risks, such as pneumothorax, barotrauma of the sinuses or ears, oxygen poisoning, and myopic shift [34]. Therefore, the indication for HBOT was commensurate with the severity of the symptoms (loss of ability to accommodate and persistent paresthesias) and also covered for the possibility that air embolism may have occurred without being detected by CT or MRI. HBOT is not indicated unless the symptoms are severe enough to warrant the risks of HBOT. In many cases, subcutaneous emphysema does not require HBOT and can be treated with alternative conservative management [35,36].

## 4. Conclusions

This case describes the formation of subcutaneous emphysema that led to pneumoperitoneum, pneumomediastinum, and cervicofacial emphysema after hip arthroscopy. This resulted in pain, numbness, swelling, and visual problems, including bilateral mydriasis and accommodation paralysis due to internal ophthalmoplegia. This was likely the result of increased stimulation of the oculosympathetic pathway, caused by irritation of the nerves responsible for dilating the pupil by pneumomediastinum and cervicofacial air. Although HBOT caused a temporary myopic shift, this resolved quickly, and HBOT helped to reverse the visual disturbances caused by the air.

## Figures and Tables

**Figure 1:**
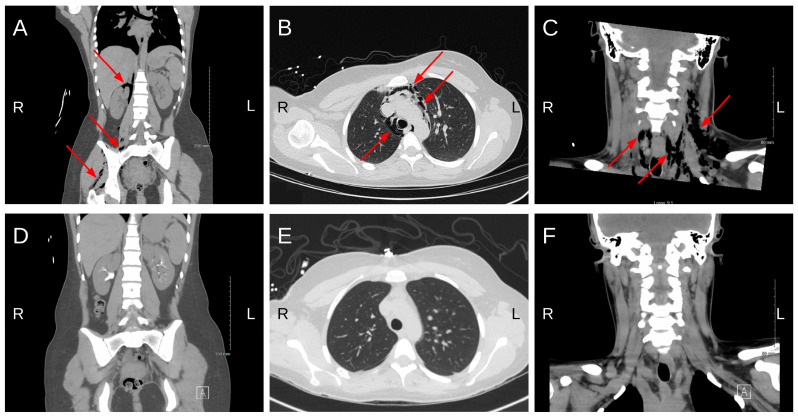
These computed tomography (CT) scans show the presence of emphysema of the (**A**) peritoneum and right hip (coronal view), (**B**) mediastinum (axial view), and (**C**) neck (coronal view) on the day of admission to the emergency department before treatment with hyperbaric oxygen. The red arrows point to the locations where subcutaneous emphysema can be seen. After two days and three hyperbaric oxygen treatments, there was reduced presence of subcutaneous emphysema in the CT scans of the (**D**) abdomen and pelvis, (**E**) chest, and (**F**) neck. The patient’s right (R) and left (L) are indicated in the scans.

**Figure 2:**
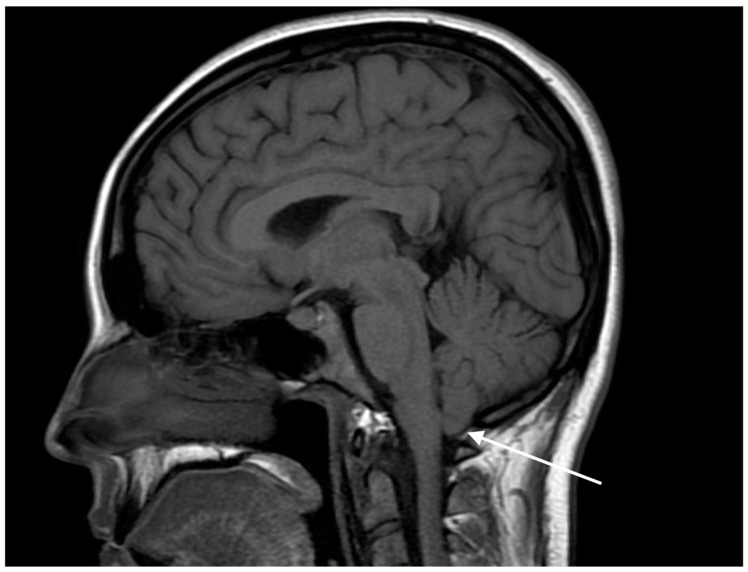
This image shows a magnetic resonance imaging slice of the brain (sagittal view), revealing a Chiari I malformation, as indicated by the white arrow, extending 7 mm below the foramen magnum.

**Figure 3:**
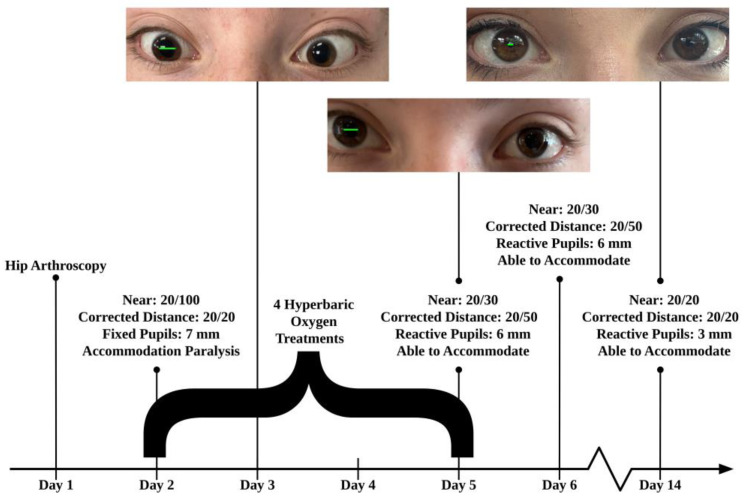
This timeline shows clinical assessment and treatment, along with photographs showing the patient’s pupil size over time. The green lines in the photographs of the eyes mark the pupil diameters.

## Data Availability

The data presented in this study are available upon reasonable request from the corresponding author to maintain patient privacy.

## Abbreviations

The following abbreviations are used in this manuscript:

CT	Computed tomography
HBOT	Hyperbaric oxygen therapy
ICP	Intracranial pressure
MRI	Magnetic resonance imaging
